# *Crataegus aronia* enhances sperm parameters and preserves testicular architecture in both control and non-alcoholic fatty liver disease-induced rats

**DOI:** 10.1080/13880209.2018.1523934

**Published:** 2018-10-30

**Authors:** Mohammad Dallak

**Affiliations:** Department of Physiology, College of Medicine, King Khalid University, Abha, Saudi Arabia

**Keywords:** Testis, testosterone, sperm, oxidative stress, high-fat diet

## Abstract

**Context:***Crataegus aronia* (syn. *Azarolus* L.) (Rosaceae) is used in traditional medicine due to its hypolipidaemic and antioxidant properties.

**Objectives:** This study investigates the effect of *C. aronia* whole plant aqueous extract on sperm parameter and testicular structure in control and non-alcoholic fatty liver disease (NAFLD)-induced rats.

**Materials and methods:** Male rats were divided into six groups (10 rats each) as control fed a standard diet (STD) (10% kcal), STD + *C. aronia* (200 mg/kg), high-fat diet (HFD) (45% kcal), HFD + *C. aronia*, HFD followed by *C. aronia*, and *C. aronia* followed by HFD. Rats were treated with *C. aronia* (once/day, orally) for four weeks.

**Results:** Compared with STD rats, STD rats co-treated with *C. aronia* had lower hepatic triglycerides (0.58 vs. 0.42 mg/g) and cholesterol (5.4 vs. 3.27 mg/g) contents, higher levels of testosterone (8.43 vs. 10.9 ng/mL), luteinizing hormone (6.05 vs. 8.1 mIU/mL) and follicle-stimulating hormone (5.8 vs. 8.0 mIU/mL) and increased epididymis weight (1.28 vs. 1.5g) and sperm count (133.2 vs. 148.3 million/0.1 mg) and motility (66.8%vs. 77.6%). They showed increased testicular levels of glutathione (6.3 vs. 7.75 µM/L) and higher protein levels of Nrf2 (0.37 vs. 0.79), γ-glutamylcysteine synthetase (0.27 vs. 0.5) and superoxide dismutase (0.92 vs. 2.1). Concomitant or post-treatment of *C. aronia* to NAFLD rats prevented the declines in sperm parameters and damage in the testis by similar effects like those found in the STD rats.

**Discussion and conclusions:** This study encourages the use of *C. aronia* in further future clinical studies.

## Introduction

The prevalence of male infertility has been increasing worldwide. Cumulative evidence has shown that alterations in sperm quantity and quality due to metabolic dysfunction play an important role in increasing male infertility rate (Hammoud et al. 2008; Du Plessis et al. 2010; Ramlau-Hansen et al. 2010; La Vignera et al. 2012; Jensen et al. 2013). Non-alcoholic fatty liver disease (NAFLD) is the most common liver clinicopathologic disease and is characterized by increased accumulation of hepatic triglyceride (TG) in patients (Chalasani et al. 2012). NAFLD occurs in the absence of excessive alcohol intake and leads to a wide spectrum of histological changes, ranging from simple steatosis to steatohepatitis (Cohen et al. 2011; Younossi et al. 2011; Chalasani et al. 2012).

Clinically and when studied experimentally, NAFLD not only is a result of metabolic changes but also plays a role in the development and progression of metabolic syndrome (Paschos and Paletas 2009). Apart from the other associated co-morbidities such as hypertension, diabetes mellitus, obesity and insulin resistance (Alwis and Day 2008; Choi et al. 2009; Targher et al. 2010), ancient and recent epidemiological, clinical and experimental studies have suggested that NAFLD impairs male reproductive function and fertility; however, the mechanisms remain unclear.

Patients with NAFLD exhibit lower serum levels of testosterone and sex hormone-binding globulin than healthy subjects of the same age (Kley et al. 1975; Völzke et al. 2010; Shin et al. 2011). A study (Li X et al. 2013) in rats has confirmed that NAFLD is an independent risk factor for reducing testosterone levels, sperm count and sperm motility. Similarly, high-fat diet (HFD)-induced NAFLD rats show delayed insemination, low testosterone levels, reduced sperm counts and motility, increased numbers of apoptotic spermatogenic cells, and altered testicular pathology (Li et al. 2013).

It is, therefore, necessary to better understand the molecular basis of NAFLD-induced reproductive impairment and develop safe strategies for reversing these adverse effects. *Crataegus aronia* (syn. *Azarolus* L) (Rosaceae) is one of the most dominant hawthorn species populating the wooded areas and mountains of the Mediterranean basin (Ali-Shtayeh et al. 2000). It is widely used in traditional medicine in the Mediterranean region for chronic disorders such as cardiovascular diseases, cancer, diabetes and sexual weakness (Ali-Shtayeh et al. 2000; Said et al. 2002; Ljubuncic et al. 2005). Like the other hawthorn species that are listed as safe herbal medication worldwide (Bahorun et al. 1994, 1996, 2003; Jayalakshmi and Devaraj 2004; Al-Hallaq et al. 2013), the pharmacological effects of *C. aronia* have been ascribed to its safe use and toleration (Al-Hallaq et al. 2013). In addition, *C. aronia* exhibits hypolipidaemic and anti-obesity properties and is a potent antioxidant for inhibiting lipid peroxidation, scavenging superoxide radicals and increasing intracellular glutathione (GSH) levels (Ljubuncic et al. 2005; Al-Hallaq et al. 2013).

Studies on other hawthorn species have shown that these species can protect against the effects of toxic drugs on male reproductive function (Jalali et al. 2011, 2012). However, despite the widespread traditional use of *C. aronia* for treating sexual problems and infertility, studies showing the effects of *C. aronia* on male reproductive function are lacking. Humayed (2017) previously showed that concomitant administration of *C. aronia* with HFD significantly improved body weight and liver indices, decreased serum lipid levels, ameliorated oxidative stress and hepatic steatosis and restored the normal liver architecture as compared to only HFD administration. In the current study, we investigate whether this effect is associated with protection against reproductive dysfunction caused by HFD-induced NAFLD.

Thus, this study has two major aims. First, we investigated the effects of *C. aronia* on male reproductive function in normal rats; second, we examined the preventative effects of *C. aronia* against reproductive dysfunction and the protective effects of *C. aronia* on male reproductive function. These factors were evaluated with respect to hormonal disturbance, oxidative stress, inflammation and apoptosis.

## Materials and methods

### Preparation of aqueous extract

A whole, dried *C. aronia* plant was purchased in January 2015 from a local pharmacognosy supplier in the Abha area of the Kingdom of Saudi Arabia (KSA). The plant originated from the mountains of Jerash, a city that is located 48 kilometres north of the capital of Jordan, Amman (Coordinates: 32°16′20.21″N 35°53′29.03″E). The collection records indicated that the plant was dried and naturally preserved for only 1 month. The plant was identified by Professor Hesham Solaiman from the Department of Pharmacognosy at the College of Pharmacy at King Khalid University (KKU), Abha, KSA. The dried whole plant (1 kg) was extracted in 1 L distilled water (*w*/*v*) in the pharmacognosy lab (KKU, Abha, KSA) as previously described (Shatoor et al. 2012; Humayed 2017). After filtration and evaporation, the final residue (32 g) was reconstituted in distilled water to a final concentration of 1 g/mL and was refrigerated until use.

### Preparation of diets

Standard diet (STD) and HFD were prepared as described by Li et al. (2013) with some modifications. The compositions of both diets are shown in Table 1. STD (100 g) contained 19.2 g protein (20% kcal), 67.3 g carbohydrate (70% kcal) and 4.3 g fat (10% kcal), providing approximately 3.85 kcal/g of fat. In contrast, 100 g of HFD contained 24 g protein (20% kcal), 40 g carbohydrate (35% kcal) and 24 g fat (45% kcal), providing approximately 4.73 kcal/g of fat. The diets and all other treatments were stored at 4 °C.

**Table 1.** Ingredient and nutrient composition of the diets.

	Low-fat diet (LFD)		High-fat diet (HFD)	
Ingredients (g/kg)	Weight	kcal	Weight	kcal
Casein	200.0	800	200.0	800
l-Cystine	3.0	12	3.0	12
Corn starch	315.0	1260	72.8	291
Maltodextrin 10	35.0	140	100.0	400
Sucrose	350.0	1400	172.8	691
Soybean oil	25.0	225	25.0	225
Lard	20.0	180	177.5	1598
Cellulose	50.0	00	50.0	00
Vitamin-mineral premix	10.0	40	10.0	40.0
Potassium citrate, 1 H2O	16.5	00	16.5	00
DiCalcium phosphate	13.0	00	13.0	00
Calcium carbonate	5.5	00	5.5	00
Mineral mix	10.0	00	10.0	00
Choline chloride	2.0	00	2.0	00
Total	1055	4057	858.15	4057
Cholesterol (mg)/4057 kcal	14.4		127.8	

* Cholesterol in lard = 0.72 mg/g.

### Animals and experimental design

All experiments and procedures in the current study were approved by the animal ethical committee of the Medical School at KKU and were in accordance with the guidelines established by the care and use of laboratory animals published by the US National Institutes of Health (NIH Publication No. 85-23, revised 1996). Healthy, adult, male Wistar rats (aged 4 weeks and weighing 90–100 g) were obtained from the animal facility. The rats were housed at 23 ± 1 °C and 55 ± 10% humidity and under a 12 h light/dark cycle and were allowed access to food and water *ad labium*. After 1 week of acclimation, the rats were randomly divided into six groups (10 rats each) and administered all treatments daily for 12 weeks. Rats were classified as follows:

1. STD group (STD): fed STD for 12 weeks.
2. STD + *C. aronia* group: fed STD and administered a concomitant dose of *C. aronia* for 8 weeks and then continued on STD for another 4 weeks.
3. NAFLD model group: fed HFD for the first 8 weeks and then returned to STD for the next 4 weeks.
4. HFD + *C. aronia* group: fed HFD and administered a concomitant dose of C. aronia for the first 8 weeks and then returned to STD for the next 4 weeks.
5. HFD then *C. aronia* group: fed HFD for the first 8 weeks and then administered *C. aronia* for the next 4 weeks.
6. *C. aronia* then HFD group: fed *C. aronia* for the first 4 weeks then administered HFD for the next 8 weeks.

In groups 2, 4, 5 and 6, *C. aronia* was orally administered at a final concentration of 200 mg/kg. This concentration has been shown to be safe, to have hypolipidaemic and anti-obesity effects (Al-Hallaq et al. 2013), and to protect the rat liver from HFD-induced NAFLD (Humayed 2017).

### Mating and pregnancy rate

During the last 2 weeks of treatment, all males in all groups were cohabitated with two proestrus females. The presence of sperm was checked in the female vaginas after flushing with normal saline. The number of days required to confirm mating was recorded. In addition, the number of pregnant females and the number of pups, with their weights at birth and 1 week after birth, were recorded.

### Blood and tissue collection

On the last day of treatment, all rats were fasted for 12 h, weighed, and anaesthetized with sodium pentobarbital (60–70 mg/kg, i.p.). Blood samples (4 mL) were collected into EDTA and plain tubes by cardiac puncture to collect plasma and sera, respectively, which were used for further biochemical analysis. All animals were ethically sacrificed, and both testes were removed. The adipose and connective tissues surrounding the testes were then removed, and the testes were weighed and washed with ice-cold phosphate-buffered saline (PBS), pH 7.4, containing 0.16 mg/mL of heparin to remove any red blood cells (erythrocytes) and clots. One testis from each rat was directly fixed in 10% buffered formalin, whereas the other was dissected into smaller parts and stored at –80 °C for further biochemical analysis. At the same time, the right epididymis obtained from each rat was collected, weighed, and freshly used for sperm count, motility and morphology analyses. Parts of the fresh livers were directly collected from all rats, stored at –80 °C, and used later to extract lipids. Other liver parts were directly placed in 10% formalin solution and used later for histological analysis.

### Biochemical analysis of the sera and tissues

Liver lipids were extracted as described by Folch (1957). The lipid contents, including total TGs, total cholesterol (CHOL), high-density lipoprotein (HDL) and low-density lipoprotein (LDL), were determined in both the sera and liver extract using commercially available colorimetric kits (HUMAN Gesellschaft für Biochemica and Diagnostica, Wiesbaden, Germany). Rat enzyme-linked immunosorbent assay kits were used to measure the serum levels of circulatory total testosterone (Cat. No. E0930Ra; Shanghai Crystal Day Biotech Co., Ltd., Shanghai, China) and luteinizing hormone (LH) (Cat. No. CSB-E12654r; Cusabio Biotech Co., Ltd., Houston, TX). Plasma glucose levels were measured using a colorimetric assay kit (Cat. No. ab65333; Abcam, Cambridge, UK). Plasma insulin levels were determined using a rat insulin enzyme-linked immunosorbent assay kit (Cat. No. ERINS; Thermo Fisher Scientific, Waltham, MA). Insulin sensitivity was determined using the homoeostasis model assessment of insulin resistance (HOMA-IR), according to the following formula: HOMA-IR = {FPG (mg/dL) × fasting plasma insulin levels (μU/mL)}/405, where FGP stands for fasting plasma sugar.

### Semen analysis

Sperm count and motility were evaluated as described in our previous study (Eleawa et al. 2014). After being minced, the right cauda epididymis from each rat was diluted 1:20 with normal saline (0.9% NaCl) and incubated for 5 min at 37 °C in a Petri dish. The total number of sperms was counted in a glass haemocytometer at 400× in the five squares of the central area. At the same time, motile and immotile sperms were counted in a total of 600 sperm fields, and the results were expressed as percentages (%). On a separate glass slide, a drop of eosin was added to the sperm suspension for morphological examination under a light microscope. The following morphologies were determined: absence of head, the absence of tails, tail-bending, tail-coiling, mid-piece curving and mid-piece bending. All procedures were performed in triplicate for each sample, and an average was determined. Data were presented as an average of 10 rats/group.

### Biochemical measurements in the testis

Parts of the frozen testis obtained from each rat were homogenized in cold phosphate buffer (pH 7.0) to obtain the homogenate supernatant, which was used to determine the reduced GSH content (Cat. No. 703002; Cayman Chemical, Ann Arbor, MI), malondialdehyde (MDA) levels (Cat. No. NWK-MDA01; Northwest Life Science Specialties, Seattle, WA) and superoxide dismutase (SOD) activity (Cat. No. 706002; Cayman Chemical). All procedures were conducted in duplicate and were performed in accordance with the manufacturer’s instructions.

### Western blotting

Whole proteins were extracted from frozen livers using a Millipore extraction kit (Cat. No. 2140; Merck Millipore, Billerica, MA) to which protease inhibitor cocktail (Cat. No. P8340; Sigma-Aldrich, St. Louis, MO) was added according to the manufacturer’s instructions. Protein concentrations in each sample were determined using a Pierce BCA protein assay kit (Cat. No. 23225; Rockford, IL). The protein samples (60 µg) were separated by 10% SDS-PAGE and were manually transferred to nitrocellulose membranes. The membranes were incubated overnight at 4 °C with primary antibodies against nuclear erythroid 2-related factor 2 (Nrf2) (Cat. No. 4399, 120 kDa, 1:500), Keap1 (Cat. No. 4678, 60-64 kDa, 1:1000), SOD-1 (Cat. No. 2770, 23 kDa, 1:1000) and β-actin (Cat. No. 4970, 45 kDa, 1:200), all of which were purchased from Cell Signaling Technology (Danvers, MA) and against γ-glutamylcysteine synthetase (γ-GCS) (Cat. No. sc-390811, 73 kDa, 1:1000; Cell signalling Biotechnology). Membranes were then washed and incubated with the corresponding secondary horseradish peroxidase-conjugated secondary antibodies. Antigen–antibody interactions were detected by chemiluminescence (Pierce ECL reagents) and quantified using C-DiGit blot scanner (LI-COR Biosciences, Lincoln, NE) using the supplied image studio DiGits software. Protein expression was presented as the relative expression compared with that of β-actin. Experiments were performed in duplicate, and data were presented as an average for six rats per group.

### Histological evaluation

Formalin preserved testes and liver sections embedded in paraffin were cut into 5 μm sections, processed, and stained with haematoxylin and eosin (H&E). All sections were examined under a light microscope by a different histologist who was unaware of the groups.

### Statistical analysis

All data were collected and analyzed by one-way analysis of variance, followed by Tukey’s *t*-test to detect significant differences between various groups. All data were expressed as the mean ± SD, and *p* < 0.05 was considered statistically significant.

## Results

### Liver pathology, plasma insulin, glucose levels and serum and hepatic lipid content

**Figure 1.**
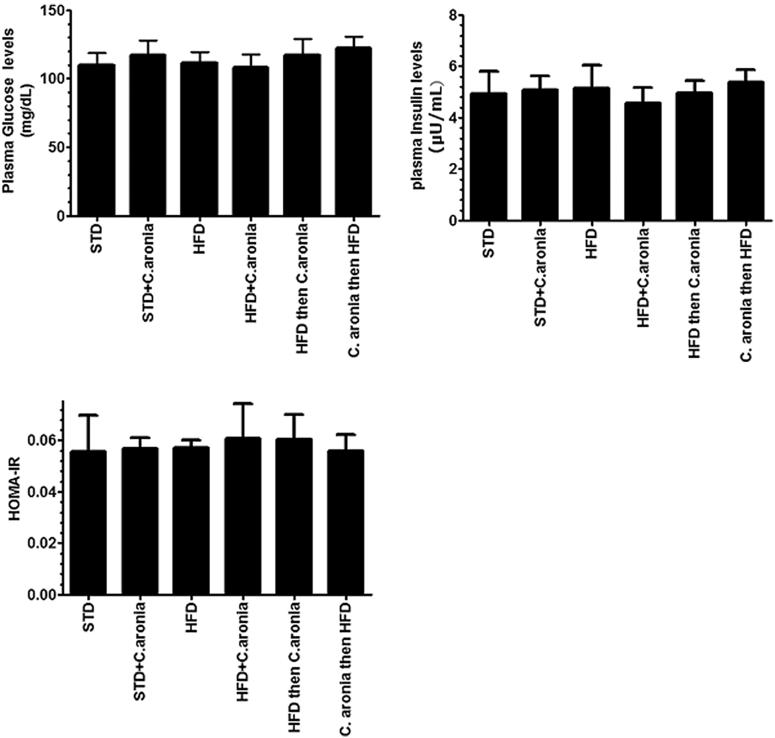
Levels of plasma glucose (A) and insulin (B) and calculated homeostasis model assessment (HOMA) in rats of all groups. Values were considered significantly different at *p* < 0.05. ^a^vs. STD; ^b^vs. STD + *C. aronia*; ^c^vs. HFD; ^d^vs. HFD + *C. aronia*; ^e^vs. HFD then *C. aronia*. STD: standard diet; HFD: high-fat diet.

**Figure 2.**
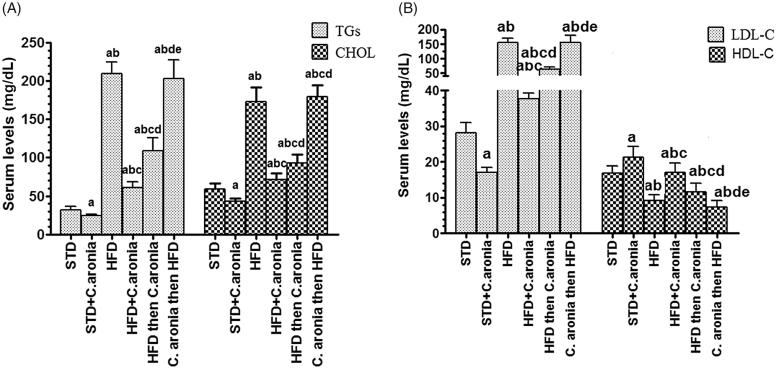
Levels of serum triglycerides (TGs), total cholesterol (CHOL), low-density lipoproteins (LDL), and high-density lipoproteins (HDL) in all groups of rats. Values are expressed as the mean ± SD for 10 rats in each group. Values were considered significantly different at *p* < 0.05. ^a^: vs. STD; ^b^: vs. STD + *C. aronia*; ^c^: vs. HFD; ^d^: vs. HFD + *C. aronia*; ^e^: vs HFD then *C. aronia*. STD: standard diet; HFD: high-fat diet.

**Figure 3.**
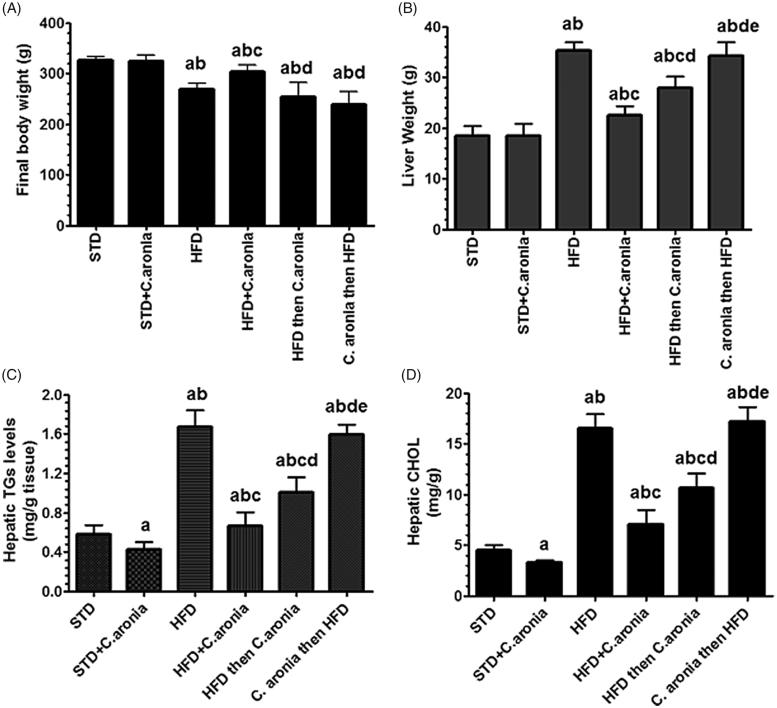
Liver and final body weights and hepatic levels of triglycerides (TGs) and total cholesterol (CHOL) in rats of all groups. Values are expressed as the mean ± SD for 10 rats in each group. Values were considered significantly different at *p* < 0.05. ^a^: vs. STD; ^b^: vs. STD + *C. aronia*; ^c^: vs. HFD; ^d^: vs. HFD + *C. aronia*; ^e^: vs HFD then *C. aronia*. STD: Standard diet; HFD: high-fat diet.

**Figure 4.**
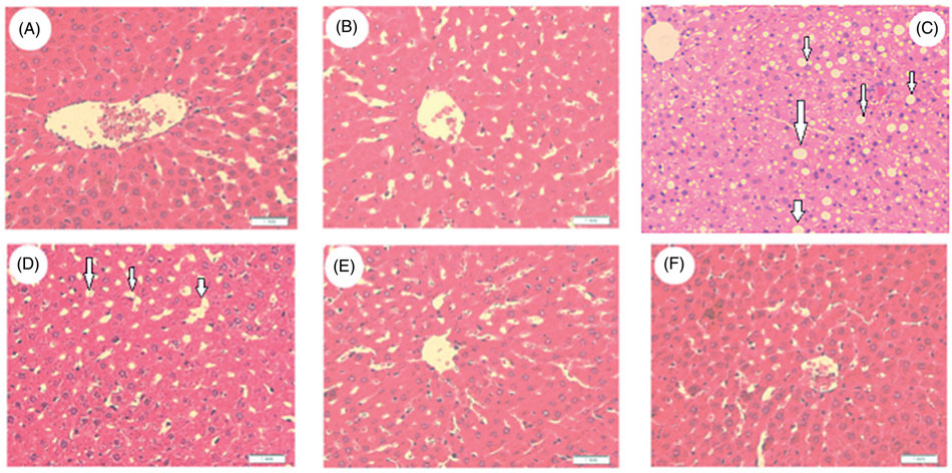
Representative images showing histology of the liver of all groups of rats. A and B were taken from STD and STD + C. aronia-treated rats, respectively. Both pictures show normal cellular arrangement, architecture, and morphology. C and D were taken from HFD and C. aronia then HFD-treated rats, respectively. Both pictures show typical NAFLD-induced steatosis where cells contain small (small white arrow) and large fat granules (large white arrows). E and F were taken from HFD + C. aronia and HFD then C. aronia-treated rats, respectively. Both pictures show few fat granules with normal cellular arrangement and morphology, as observed in the STD group. STD: standard diet; HFD: high-fat diet. H&E stain, 200×.

Plasma insulin and glucose levels, as well as the calculated HOMA-IR, did not change in any group following the various treatments (Figure 1). Compared with control rats fed STD, STD + *C. aronia*-fed rats had significantly lower serum and hepatic levels of TGs, CHOL and serum LDL (*p* < 0.05) and significantly higher levels of serum HDL with normal liver architectures (*p* < 0.023) (Figures 1 and 2). However, opposite trends were showed by NAFLD model rats and *C. aronia* followed by HFD-fed rats, with higher serum and hepatic levels of TGs, CHOL and LDL (*p* < 0.001) and concomitantly lower serum HDL levels (*p* < 0.01). In addition, their livers showed cytoplasmic lipid droplets of various sizes, indicating the development of hepatic steatosis (Figures 2–4). In contrast, HFD followed by *C. aronia*-treated rats had partially, but significantly, improved serum levels and hepatic lipid contents, approaching the normal levels observed in STD rats (*p* < 0.05), and their livers showed a reduced number of cytoplasmic fat droplets. Rats fed with HFD + *C. aronia* showed the maximum improvements in all of these parameters (Figures 2–4).

### Sex organ weights, sperm quantity, morphology and motility, and mating outcome

**Figure 5.**
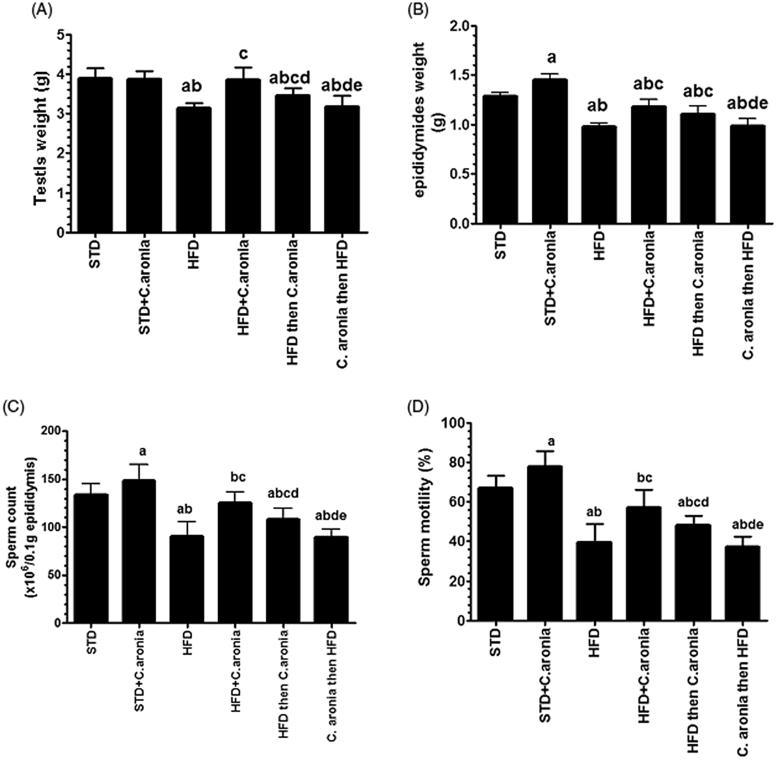
Average testis weights (A), epididymis weights (B), sperm count (C) and percentages of motility (D) in all groups of rats. Values are expressed as the mean ± SD for 10 rats in each group. Values were considered significantly different at *p* < 0.05. ^a^vs. STD; ^b^vs. STD + *C. aronia*; ^c^vs. HFD; ^d^vs. HFD + *C. aronia*; ^e^vs. HFD then *C. aronia*. STD: standard diet; HFD: high-fat diet.

STD rats co-administered with *C. aronia* showed significantly increased epididymis weight (*p* < 0.022) and increased sperm motility (*p* < 0.041) and count (*p* < 0.032). Their testicular weights were unaltered and their sperm morphology and total abnormalities were unaffected (Figure 5 and Table 2). In addition, the infertility index and birth weights were unaffected, though the number of pups at birth was higher (Table 3) than those of STD-fed rats. NAFLD model rats and *C. aronia* then HFD-fed rats showed similar a significant decrease in testis and epididymis weights, sperm count and motility (Figure 5), fertility index and the number of pups at birth *(p* < 0.05) (Table 3), compared with STD-fed rats. Sperm from these rats showed significantly higher percentages of coiled and tail-less sperm *(p* < 0.001) (Table 2). In contrast, compared to NAFLD model rats, both *C. aronia* then HFD and HFD + *C. aronia*-fed rats had significantly higher testicular and epididymal weights, increased sperm count and motility, and lower percentages of tail-less and coiled sperm *(p* < 0.05) (Figure 5 and Table 2), concomitant with an increased fertility index and number of pups at birth (Table 3). However, analysis of variance showed that improvements in the levels of all parameters were significantly higher when the extract was concomitantly administered with HFD rather than as a post-treatment dose.

**Table 2.** Characterization of epididymal sperm morphology in groups of rat (%).

Group	Absence of tail		Absence of head	Tail bending	Tail coiling	Midpiece pending	Total abnormality
STD	1.31 ± 0.2	1.11 ± 0.2	0.92 ± 0.03	1.11 ± 0.07	1.42 ± 0.1	5.8	
STD + *C. aronia*	1.41 ± 0.09		1.11 ± 0.14	0.63 ± 0.03	1.21 ± 0.12	1.24 ± 0.2	5.03
HFD	7.82 ± 1.6^ab^		1.31 ± 0.08	0.81 ± 0.02	13.10 ± 2.1^abc^	1.10 ± 0.2	23.38^abc^
HFD + *C. aronia*	2.87 ± 0.62^abc^		1.11 ± 0.3	0.73 ± 0.03	5.80 ± 0.5^abc^	1.20 ± 0.3	11.71^abc^
HFD then *C. aronia*	4.34 ± 0.75^abcd^		1.14 ± 0.1	0.68 ± 0.02	8.10 ± 1.5^abcd^	1.32 ± 0.3^abc^	15.56^abcd^
*C. aronia* then HFD	6.91 ± 1.2^abde^		1.23 ± 0.3	0.78 ± 0.02	14.33 ± 2.4^abde^	1.10 ± .3	24.32^abde^

Values are expressed as mean ± SD. *N* = 10 per group. Values are statistically significant at *p* < 0.05.

^a^: vs. STD; ^b^: vs. STD + *C. aronia*; ^c^: vs. HFD; ^d^: vs. HFD + *C. aronia*; ^e^: vs. HFD then *C. aronia*. STD: standard diet; HFD: high-fat diet.

**Table 3.** Mating outcome in all groups of rats.

Group	No. of females	No. of Pregnant females	Pregnancy index	No. of pubs at birth	Weight of pubs at birth	Survival at day 7	Weights of pubs at day 7
STD	20	20	100%	155	6.50 ± 0.86	151/155 (97.4%)	12.1 ± 1.6
STD + *C. aronia*	20	20	100%	164	6.45 ± 0.45	159/164 (96.9%)	11.9 ± 1.4
HFD	20	16	80%	114	6.75 ± 32	110/114 (96.4%)	11.8 ± 1.4
HFD + *C. aronia*	20	20	100%	136	6.56 ± 0.65	132/136 (97.0%)	12.3 ± 1.2
HFD then *C. aronia*	20	18	90%	127	6.66 ± 0.54	122/127 (96.1%)	11.9 ± 1.9
*C. aronia* then HFD	20	14	70%	118	6.43 ± 0.34	116/118 (98.3%)	11.4 ± 1.4

Values are expressed as mean ± SD. *N* = 10 per group.

Values are statistically significant at *p* < 0.05.

^a^vs. STD; ^b^vs. STD + *C. aronia*; ^c^vs. HFD; ^d^vs. HFD + *C. aronia*; ^e^vs. HFD then *C. aronia*. STD: standard diet; HFD: high-fat diet.

### Sex hormone levels

**Figure 6.**
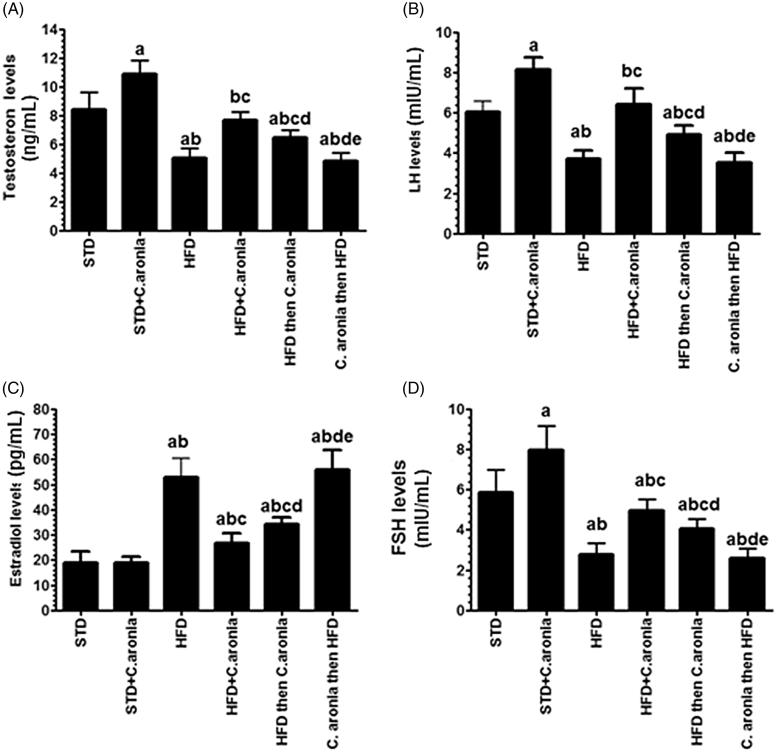
Levels of circulatory testosterone (A), luteinizing hormone (LH, B), oestradiol (C) and follicle-stimulating hormone (FSH, D) in the sera of allgroups of rats. Values were considered significantly different at *p* < 0.05. ^a^vs. STD; ^b^vs. STD + *C. aronia*; ^c^vs. HFD; ^d^vs. HFD + *C. aronia*; ^e^vs. HFD then *C. aronia*. STD: standard diet; HFD: high-fat diet.

Circulatory oestradiol levels were not significantly changed, while circulatory testosterone, follicle-stimulating hormone, and LH were significantly increased in the sera of STD-fed rats (*p* < 0.05) (Figure 6). Compared with control rats, NAFLD model rats and *C. aronia* then HFD-fed rats showed significantly lower circulatory levels of testosterone, follicle-stimulating hormone (FSH) and LH and a parallel increase in the circulatory levels of oestradiol (*p* < 0.01) (Figure 6). However, HFD + *C. aronia*-fed rats and HFD then *C. aronia*-fed showed significantly lower circulatory oestradiol levels and higher circulatory levels of testosterone, FSH, and LH in the sera (*p* < 0.05). The improvements were more significant when the extract was administered as a conjugate with HFD (Figure 6).

### Levels of oxidative stress in testicular homogenates

**Figure 7.**
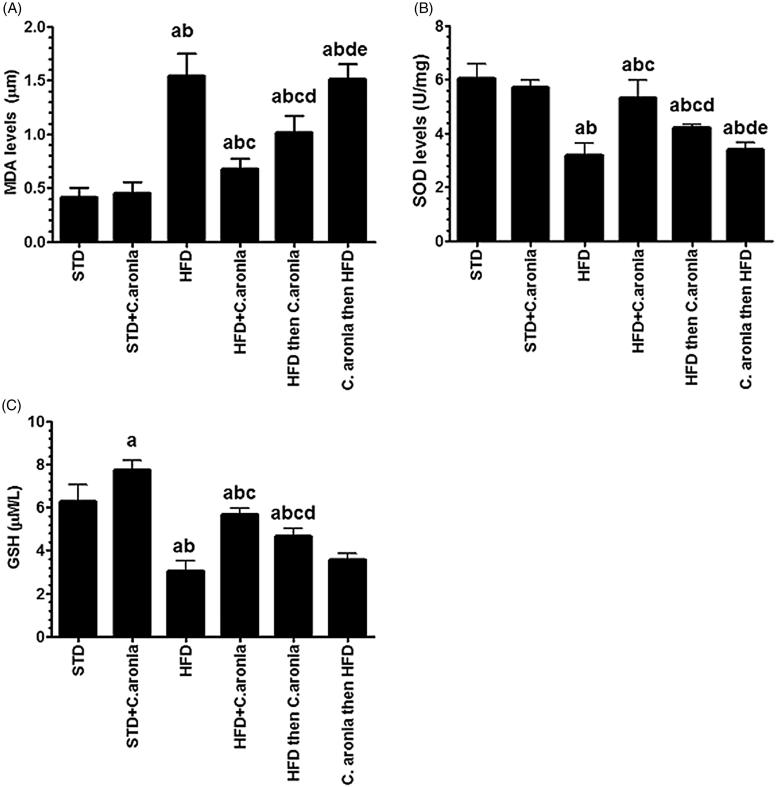
Levels malondialdehyde (MDA, A) and glutathione (GSH, C) and activities of superoxide dismutase (SOD, B) in the testis homogenates of all groups of rats. Values were considered significantly different at *p* < 0.05. ^a^vs. STD; ^b^vs. STD + *C. aronia*; ^c^vs. HFD; ^d^vs. HFD + *C. aronia*; ^e^vs. HFD then *C. aronia*. STD: standard diet; HFD: high-fat diet.

The testicular levels of MDA and GSH and the activity of SOD are shown in Figure 7. Among all samples, GSH levels were significantly increased (*p* < 0.028) in the testicular homogenates of only the STD + *C. aronia*-fed rats, compared to the control rats fed STD alone. Significant and similar increases in MDA levels with parallel decreases in SOD activities and GSH levels (*p* < 0.01) were observed in the testicular homogenates of the NAFLD model rats and *C. aronia* then HFD-fed rats. In contrast, HFD then *C. aronia*-fed rats showed significant decreases in MDA levels and significant increases in GSH levels and SOD activities (*p* < 0.01). However, a high decrease in MDA levels with high increases in GSH levels and SOD activities was detected in HFD + *C. aronia*-fed rats compared to NAFLD model rats (*p* < 0.001) (Figure 7).

### Alteration in protein levels of antioxidant markers

**Figure 8.**
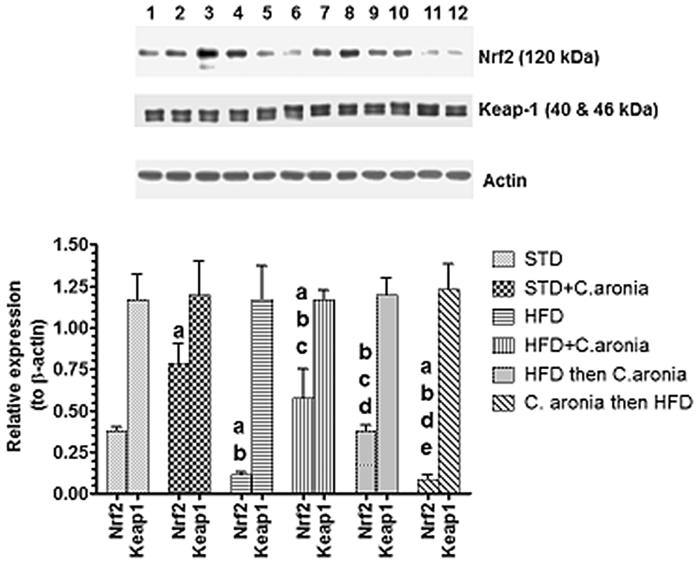
Protein levels of Nrf2 and Keap1 in the testis of all groups of rats. Values were considered significantly different at *p* < 0.05. ^a^vs. STD; ^b^vs. STD + *C. aronia*; ^c^vs. HFD; ^d^vs. HFD + *C. aronia*; ^e^vs. HFD then *C. aronia*. STD: standard diet; HFD: high-fat diet.

**Figure 9.**
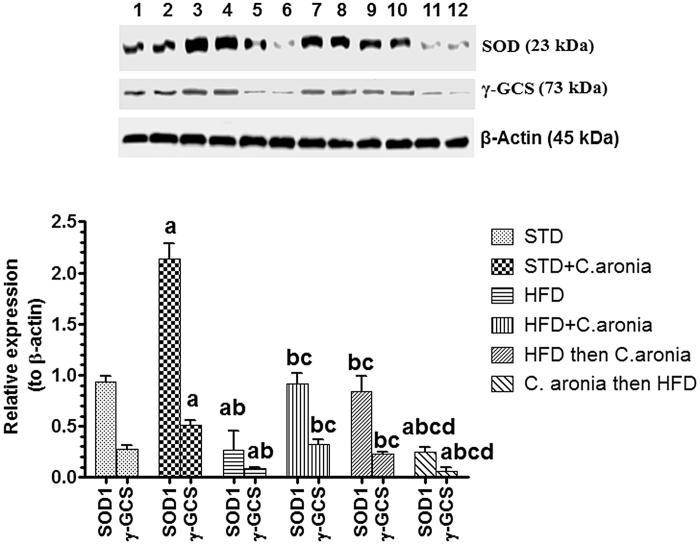
Protein levels of superoxide dismutase-1 (SOD-1) and γ-glutamylcysteine synthetase (γ-GCS) in the testis of all groups of rats. Values were considered significantly different at *p* < 0.05. ^a^vs. STD; ^b^vs. STD + *C. aronia*; ^c^vs. HFD; ^d^vs. HFD + *C. aronia*; ^e^vs. HFD then *C. aronia*. STD: standard diet; HFD: high-fat diet.

The protein levels of Nrf2, Keap1, SOD-1 and γ-GCS are shown in Figures 8 and 9. The sizes of all targeted proteins were similar to the expected levels. The protein levels of all the above proteins were normalized to their corresponding expression of β-actin in the same rat. Significant elevations in levels of Nrf2, SOD and γ-GCS, with no alterations in the levels of Keap1, were detected in STD + *C. aronia*-fed rats compared to those in STD-fed rats. However, significant decreases in Nrf2, SOD and γ-GCS, with stable Keap1 expression, were detected in the testis of NAFLD model rats that received the vehicle or were pre-treated with *C. aronia* (*p* < 0.05). In contrast, significantly increased Nrf2, SOD and γ-GCS protein levels, with no changes in Keap1 expression, were observed in the testis of HFD + *C. aronia* or HFD then *C. aronia*-fed rats, as compared with NAFLD model rats (Figure 8).

### Testis microscopic changes

**Figure 10.**
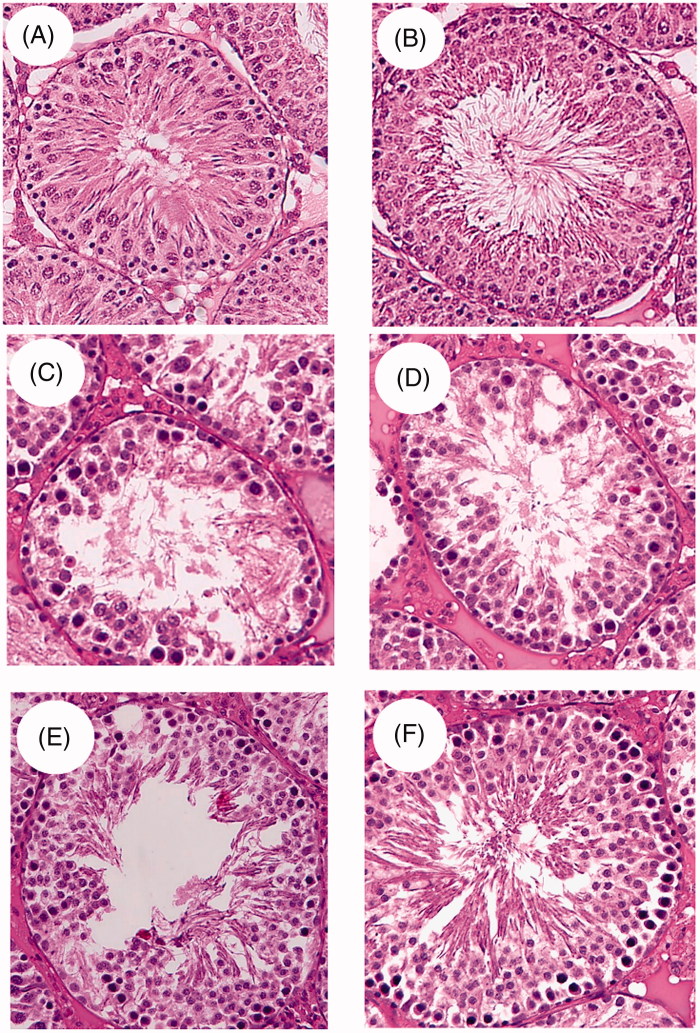
Photomicrographs of histopathological changes seen in the testis of all groups of rats. A was taken from an STD rat and showed the normal testicular architecture, preserved seminiferous tubules with the preserved definite membrane, and small lumen filled with mature sperm. All testicular cells including spermatogonia, primary spermatocytes, early and late spermatids, Sertoli cells and Leydig cells were abundant and well preserved. B was taken from an STD + *C. aronia* rat and showed similar features to those seen in the STD rat but with a wider and better preserved seminiferous tubule and more mature sperm in the centre. C and D were taken from an HFD and *C. aronia*-treated rat where both pictures show abnormal testicular structures with clear vaculations and swelling in most germ cells, loss of Sertoli cells, and a reduced number of mature sperm. E was taken from an HFD then *C. aronia*-treated rat and showed less swelling in the germ cells and regeneration of the Sertoli cell with fewer vacuoles. F was taken from an HFD + *C. aronia*-treated rat showing nearly normal testicular morphology with normal seminiferous tubule structure. All germs cells were observed and mature sperms were also dominant in the centre of the tube. However, some swelling and detachments in spermatogonia cells were observed in some sections.

Seminiferous tubules obtained from STD or STD + *C. aronia*-fed rats were well differentiated, and each had a preserved definite membrane with a small lumen filled with sperm. All spermatogenic cells (spermatogonia, primary spermatocytes, early spermatids, late spermatids and Sertoli cells) were abundant and well preserved (Figure 10(A,B)). However, although the basement membrane of the seminiferous tubule was intact, the testes obtained from NAFLD rats showed abnormal testicular structures, with clear vacuolation and swelling in most germ cells, loss of Sertoli cells, and a reduced number of mature sperm (Figure 10(C,D)). In contrast, morphological assessment of the testes obtained from NAFLD rats post-treated with *C. aronia* showed greater improvements in the structure of the seminiferous tubule, with less swelling in the germ cells, less regeneration of the Sertoli cells, and few vacuoles. However, the sperm count appeared to be reduced in some tubules (Figure 10(E)). Normal testicular morphology and seminiferous tubule structures were observed in the HFD + *C. aronia*-fed rats, where mature sperm was dominant in the centres, germ cells were detected, and the number of Sertoli cells was increased. However, some swelling and detachments in spermatogonia cells were observed in some sections (Figure 10(E)).

## Discussion

The findings of this study demonstrate the safety and efficiency of *C. aronia* aqueous extract on male rat reproductive function in both control and NAFLD-induced animals, supporting the traditional use of this plant to treat sexual dysfunction. In this study, *C. aronia* enhanced or restored reproductive function, sperm parameters, and fertility output, in both control and NAFLD-induced male rats through its hypolipidaemic effects, increasing circulatory testosterone levels and testicular endogenous antioxidant potential. The latter mechanism was shown to be mediated by the activation of Nrf2 and its downstream antioxidant defence proteins, SOD and γ-GCS.

Obesity, diabetes mellitus and/or ischaemia-reperfusion injury can adversely affect male fertility by lowering serum testosterone levels and ejaculate volume and altering sperm parameters (La Vignera et al. 2012; Li et al. 2013; Navarro-Casado et al. 2010; Rao et al. 2013). Thus, it is challenging to validate the NAFLD model to exclude the side effects of all other co-morbidities. Because HFD-fed rats had hepatic steatosis and showed significant increases in the levels of serum TGs, CHOL and LDL with no changes in serum glucose, insulin and HOMA-IR levels, the adverse effects of HFD on male reproductive function seen in this study are likely to be related to the development of NAFLD. Based on these data, we validated our model and continued treatments in all other groups.

Testosterone, which is crucial for spermatogenesis, is synthesized and secreted by the interstitial cells of Leydig in the testes following stimulation by LH (Blanco-Rodriguez and Martinez-Garcia 1998). In addition, FSH acts within the tubules to elevate the number and function of Sertoli cells, which facilitate the progression of germ cells to spermatozoa and indirectly regulate spermatogenesis (Eleawa et al. 2014). In the current study, the administration of *C. aronia* to control rats for 4 weeks significantly elevated epididymal weights and serum levels of testosterone, FSH and LH, without altering oestradiol level. This was associated with the normal sperm total abnormality percentage and significant increases in sperm count and motility, as well as an increased number of pups at birth. In addition, less time was needed for cohabitation. Preserved testicular structure, seminiferous tubules and germ cell morphologies were observed in this group of rats.

These findings suggest a stimulatory effect of *C. aronia* on testosterone production and reproductive outcomes. Oestradiol is a potent stimulator of the synthesis and secretion of the gonadotropin-releasing hormone, LH and FSH (Pentikäinen et al. 2000). The stable levels of oestradiol in the serum of these rats with significant increases in LH and FSH suggest that *C. aronia* directly affected the hypothalamus–pituitary axis (HPA).

Oxidative stress is a known independent factor that reduces sperm count, motility and viability, lowers fertilization abilities and initiates germ cells apoptosis (Wang et al. 2003). Compared with the effects in control rats, administration of *C. aronia* extract significantly enhanced basal testicular GSH levels and lowered levels of MDA with no alterations in SOD activity, suggesting a potential to increase the endogenous antioxidant potential in rat testes. This was further supported by the increased levels of SOD and γ-GCS, the major enzyme responsible for GSH synthesis. These findings are in accordance with those of Ljubuncic et al. (2005), who showed that *C. aronia* enhances GSH levels in cultured HepG2 cells *in vitro*, leading to enhanced circulatory testosterone levels. Furthermore, serum TGs markedly suppressed Leydig cell survival and function to produce testosterone in a time- and dose-dependent manner *in vitro*. Interestingly, administration of *C. aronia* significantly decreased the hepatic and serum levels of TGs and CHOL of control rats, suggesting that this extract has potent hypolipidaemic effects that impact Leydig cell differentiation and another mechanism for increasing circulatory testosterone levels. In support of this, Al-Hallaq et al. (2013) and Humayed (2017) showed that the levels of TGs and CHOL were significantly reduced in the sera and livers of control rats treated with *C. aronia*.

In contrast and in accordance with many reports (Völzke et al. 2010; Shin et al. 2011; Li et al. 2013; Lia et al. 2015), NAFLD model rats showed significantly lower serum testosterone levels, sperm counts and sperm motility, with parallel increases in the percentages of sperm abnormalities, prolonged cohabitation periods, inhibited fertility index, and decreased numbers of pups at birth. In addition, HFD lowered the levels of FSH and LH and reduced testicular weights, all of which are consistent with the findings of Li et al. (2013). Such decreases in testicular weight may be related to the degradation of structural proteins (Rajkumar et al. 1991), low testosterone levels, impaired spermatogenesis and increased germ cell apoptosis (Katoh et al. 2002; Prahalathan et al. 2004; Pandya et al. 2012).

In general, low testosterone levels are one of the major mechanisms by which NAFLD reduces male fertility output (Li et al. 2013). Independent of any other comorbidities, mechanisms for lowering testosterone levels in NAFLD patients or animals include high circulatory TGs, decreased circulatory LH levels, low mRNA and protein levels of testicular steroidogenic acute regulatory protein and hepatic sex hormone-binding globulin levels, increased testicular temperature, high testicular inflammation and low numbers of spermatogenic and Leydig cells (Shin et al. 2011; Li et al. 2013; Lia et al. 2015).

We also observed low levels of oestradiol levels, which have been shown to inhibit the apoptosis of sperm cells and enhance the stimulation of HPA (Pentikäinen et al. 2000), in the sera of NAFLD model rats. This finding suggests a new mechanism by which NAFLD lowers testosterone, LH and FSH levels. In addition, NAFLD exaggerated the oxidative stress response as evidenced by the lowered testicular levels of GSH and SOD activity, leading to significant alterations in the histopathology of the rat testes, an effect that has been described in various other studies on HFD-fed animals (Hammoud et al. 2008; Du Plessis et al. 2010; Alhashem et al. 2014).

In regard to hepatic steatosis, hyperlipidaemia, and reproductive function, only when administered as a concomitant dose or after induction of NAFLD, *C. aronia* significantly reversed all measured biochemical endpoints, enhanced the fertility index, improved circulatory sex hormone levels and sperm parameter including count, motility and morphology, and ameliorated NAFLD-induced testicular histological alterations in the treated rats. Our data clearly show that *C. aronia* improved male reproductive function and sperm parameters in control and NAFLD-induced rats by 1) inducing LH, FSH and testosterone synthesis and secretion, 2) enhancing testicular GSH levels and 3) lowering hepatic TGs levels.

Based on these data, we also examined the mechanisms by which *C. aronia* enhanced testicular antioxidant potential, especially those responsible for the increases in GSH levels and SOD activity, with parallel increases in SOD and γ-GCS protein levels. For this, we targeted the Nrf2/Keap1 signalling pathway because of its crucial role as an upstream regulator of the endogenous antioxidant system in most tissues including the testes (He et al. 2018).

Under normal conditions, Nrf2 interacts with Keap1 and remains localized in the cytoplasm where it is degraded by the ubiquitin-proteasome pathway (Chen et al. 2015). However, under oxidative stress conditions, Nrf2 is rapidly phosphorylated, dissociated from Keap1, and translocated to the nucleus, where it binds to antioxidant response element sequences and along with other nuclear proteins enhances the transcription of various antioxidant genes including those leading to transcription of SOD, γ-GCS and so GSH, and haeme oxygenase-1 (HO-1) (Chen et al. 2015; Espinosa-Diez et al. 2015; Zhang et al. 2015).

Nrf2-null mice suffered from severe liver damage compared to wild-type mice (Tanaka et al. 2008). Interestingly, studies have shown that HFD either increases or decreases hepatic Nrf2 (Kim et al. 2004; Wang et al. 2010; Gupte et al. 2013; Guo et al. 2017). However, little is known about the expression of Nrf2/Keap1 in the testes of animals with NAFLD or after *C. aronia* treatment, which were examined in this study. In accordance with previous reports (Kim et al. 2004; Gupte et al. 2013), we found significant decreases in Nrf2, with no alterations in Keap1 levels in the testes of NAFLD model rats. Interestingly, Nrf2 levels were significantly increased with no alterations in Keap1 levels in the testicular tissues of control or NAFLD rats administered *C. aronia*. Being the upstream regulator, the significant increases in the levels of Nrf2 simply explains the significant increases in the testicular enzymatic activity and protein levels of SOD-1 and with significant increases in the protein levels of γ-GCS and levels of GSH.

These findings are expected, given the abundant phytochemical, polyphenol, terpene and flavonoid contents of the hawthorn species. In general, these plant-derived molecules activate Nrf2 signalling in various tissues and cell lines, even in the absence of exogenous oxidative stress stimuli (Joung et al. 2007; Tanaka et al. 2008; Chen et al. 2011; Korenor et al. 2013; Krajka-Kuźniak et al. 2013; Tanigawa et al. 2007; Jun et al. 2014). This explains the induction of Nrf2 expression in the testes of control rats. In agreement with our results, *C. pinnatifida*, another hawthorn species, induced Nrf2/HO-1 expression in ovariectomized rats (Yoo et al. 2016).

A limitation of this study is that we did not determine the active ingredients of *C. aronia*. In general, similar with other hawthorn species, *C. aronia* shows a high content of the following (fully reviewed by Edwards et al. in 2012):

1. Proanthocyanidins such as hydroxycinnamic acids, chlorogenic and ferulic acids and lignans
2. Flavonoids, such as flavonol-*O*-glycoside 2 and quercetin-3-*O*-galactoside, and flavone-C glycosides 3, vitexin 2″-*O*-rhamnoside (VOR), acetylvitexin-2″-*O*-rhamnoside and quercetin (QUR)
3. Sugars and sugar alcohols such as malic, citric, succinic, ascorbic, tartaric, quinic, protocatechuic, 3- and 4-hydroxybenzoic, salicylic and syringic acids.
4. Terpenes.

In addition to the effects on Nrf2/Keap1 expression described above, hydroxycinnamic acids, chlorogenic acid, proanthocyanidin and VOR are reactive oxygen species, which are molecular scavengers of both superoxide and hydrogen peroxide radicals and can be safely used over a wide range of doses (Petkov 1979; Bahorun et al. 1996; Chen and Ho 1997; Olthof et al. 2001; Yeh and Yen 2003; Rice-Evans 2006; Orhan et al. 2007; Edwards et al. 2012; Meng et al. 2013; Wei et al. 2014). In addition, the flavonoid VOR and chlorogenic acid are well characterized by their potent hypolipidaemic effects and can ameliorate and treat NAFLD (Meng et al. 2013; Chang et al. 2005). QUR has hypolipidaemic, antioxidant and anti-inflammatory properties against NAFLD (Li et al. 2013; Pisonero-Vaquero et al. 2015; Salomone et al. 2016; Porras et al. 2017).

In conclusion, this is the first study to determine the stimulatory effects of *C. aronia* on circulatory testosterone levels and male reproductive function and clearly demonstrate a protective and ameliorative effect of *C. aronia* NAFLD-induced reduction in male reproductive function. This effect is mediated by multiple mechanisms including the hypolipidaemic and antioxidant effect of *C. aronia,* as well as its possible direct stimulatory effect on HPA.
